# The crosstalk between microbial sensors ELMO1 and NOD2 shape intestinal immune responses

**DOI:** 10.1080/21505594.2023.2171690

**Published:** 2023-02-19

**Authors:** Aditi Sharma, Sajan Chandrangadhan Achi, Stella-Rita Ibeawuchi, Mahitha Shree Anandachar, Hobie Gementera, Uddeep Chaudhury, Fatima Usmani, Kevin Vega, Ibrahim M Sayed, Soumita Das

**Affiliations:** a Department of Pathology, University of California San Diego; San Diego, California, USA; b Department of Medical Microbiology and Immunology, Faculty of Medicine, Assiut University, Assiut, Egypt; c Department of Biomedical and Nutritional Science, University of Massachusetts-Lowell, Lowell, USA

**Keywords:** Microbial sensors, NOD2, bacterial engulfment, *AIEC*-LF82, epithelial cells and macrophages, 3D-organoid, ELMO-1

## Abstract

Microbial sensors play an essential role in maintaining cellular homoeostasis. Our knowledge is limited on how microbial sensing helps in differential immune response and its link to inflammatory diseases. Recently we have confirmed that ELMO1 (Engulfment and Cell Motility Protein-1) present in cytosol is involved in pathogen sensing, engulfment, and intestinal inflammation. Here, we show that ELMO1 interacts with another sensor, NOD2 (Nucleotide-binding oligomerization domain-containing protein 2), that recognizes bacterial cell wall component muramyl dipeptide (MDP). The polymorphism of NOD2 is linked to Crohn’s disease (CD) pathogenesis. Interestingly, we found that overexpression of ELMO1 and mutant NOD2 (L1007fs) were not able to clear the CD-associated adherent invasive *E. coli (AIEC*-LF82). The functional implications of ELMO1-NOD2 interaction in epithelial cells were evaluated by using enteroid-derived monolayers (EDMs) from ELMO1 and NOD2 KO mice. Subsequently we also assessed the immune response in J774 macrophages depleted of either ELMO1 or NOD2 or both. The infection of murine EDMs with *AIEC*-LF82 showed higher bacterial load in ELMO1-KO, NOD2 KO EDMs, and ELMO1 KO EDMs treated with NOD2 inhibitors. The murine macrophage cells showed that the downregulation of ELMO1 and NOD2 is associated with impaired bacterial clearance that is linked to reduce pro-inflammatory cytokines and reactive oxygen species. Our results indicated that the crosstalk between microbial sensors in enteric infection and inflammatory diseases impacts the fate of the bacterial load and disease pathogenesis.

## Introduction

The epithelial lining of the gastrointestinal tract harbours a plethora of microbes which include bacteria, fungi, viruses, and protozoa [1]. Although these microbes are separated by biophysical and biochemical barriers, they are in constant interaction with the epithelial cells and host immune system. Any changes in the composition of the microbes lead to perturbations in the barrier and can disrupt intestinal homoeostasis which has been recently considered as the cause of various diseases [1,2]. Intestinal epithelium plays a critical role in maintaining homoeostasis and is involved in constant sampling of the intestinal microenvironment, sensing of commensals and pathogens, secretion of compounds, and triggering immune response which influences the microbial colonization [3,4].

Detection of pathogens is essential for the host immune system to elicit antimicrobial defence mechanisms. It has been established that pattern recognition receptors (PRRs) are involved in microbial sensing and can distinguish between commensals and pathogens by identifying pathogen-associated molecular patterns (PAMPs) associated with microbes [5]. ELMO1 facilitates bacterial internalization, mounts inflammatory response, and coordinates bacterial clearance [6–9]. The ELMO1 interacting PRR, Brain Angiogenesis Inhibitor 1 (BAI1) identifies Gram negative bacteria and triggers immune response in an ELMO1-dependent manner [10]. Studies from our group have revealed that ELMO1 is involved in the sensing of microbes associated with Inflammatory Bowel Disease (IBD) and pro-inflammatory cytokines secretion [11]. Using enteroid-derived monolayers (EDMs) from the organoids isolated from colonic biopsies of IBD patients we have shown the putative role of ELMO1 in triggering the inflammatory cascade of IBD [11]. Interestingly, we found that ELMO1 induces differential immune responses between pathogens and commensals by interacting with several bacterial effectors containing the WxxxE motif [9].

Nucleotide-binding oligomerization domain-containing protein 2 (NOD2) is a cytosolic PRR belonging to the Nod-like receptor (NLR) family that recognizes processed muramyl dipeptide (MDP), the bacterial cell wall component, from both Gram-positive and Gram-negative bacteria [12]. Downstream signalling of NOD2 includes recruitment of RICK (RIP-like interacting CLARP kinase)/RIP2 (Receptor-Interacting Protein 2) and activation of nuclear factor-κB (NF-κB) and mitogen-activated protein kinase (MAPK) pathways. Consequently, the stimulation of NOD2 results in secretion of proinflammatory cytokines such as interleukin 8 (IL-8) and interleukin 1β (IL-1β), induction of autophagy, production of antimicrobial peptides, and maintaining the intestinal homoeostasis [12–15]. NOD2 plays a crucial role in the regulation of intestinal microbiota [16,17]. Interestingly, murine studies have reported that NOD2 deficient mice had a reduced capability to prevent colonization of pathogenic microbes in the intestine and had impaired bactericidal activity [18]. Several studies have identified a significant role for NOD2 in IBD especially Crohn’s Disease (CD). Mutations in *NOD2* and variants of *NOD2* have been shown to increase susceptibility to CD [19,20]. Single nucleotide polymorphisms (SNPs) and variation in NOD2 receptors were recorded in CD patients, specifically two missense mutations, R702W and G908R, and one frameshift mutation, L1007fs [19,20]. NOD2 variants associated with CD has been shown to be defective in the recognition of MDP [21]. The mutations of *NOD2* associated with CD are accompanied by impaired initiation of autophagy and bacterial elimination. However, the mechanisms through which *NOD2* mutations lead to enhanced inflammation are not completely understood.

ELMO1 and NOD2 are cytosolic sensors and are also implicated in IBD, however whether these sensors interact with each other and the influence of such interaction on bacterial pathogenesis are not known. In this study, we assessed if NOD2 and ELMO1 could interact with each other directly or indirectly to regulate the bacterial sensing. We used stem-cell based approaches to recapitulate normal gut physiology, and intestinal bacteria, *Adherent invasive E. coli* (*AIEC*-LF82), as stressor to assess gut function. We found that both these cytosolic proteins interact with each other and regulate bacterial load, reactive oxygen species (ROS) generation and induction of pro-inflammatory cytokine response in macrophages and gut epithelial cells. Overall, we found that the interactions of these two bacterial sensors play an important role in bacterial sensing and inflammation during enteric infections of *AIEC*-LF82 and *Salmonella*.

## Results

### ELMO1 interacts with cytosolic microbial sensor NOD2

**Figure 1.**
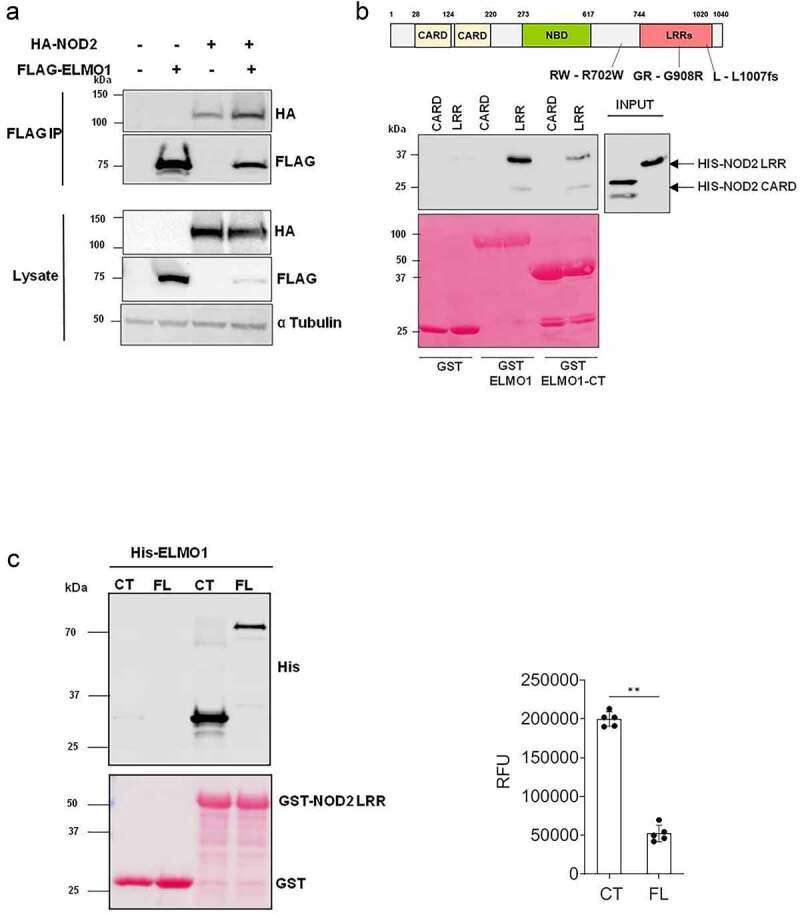
ELMO1 binds the LRR domain of NOD2 through the C terminal end. **a**. HEK293 cells were co-transfected with Flag-ELMO1 and HA-NOD2. After transfections, cells were lysed, normalized for protein content and precipitated using anti-FLAG antibody. Immunoprecipitants and cell lysates were visualized by immunoblotting with corresponding antibodies. **b**. the schematic shows the structure of NOD2 protein and associated mutations involved in IBD. To detect the regions of NOD2 that bind ELMO1, Pulldown assay using GST, GST-ELMO1 full length (FL) and GST-ELMO1 C-terminal (CT) were immobilized on glutathione beads. The soluble recombinant his-NOD2 CARD or his-NOD2 LRR proteins were incubated with the beads. Bound NOD2 proteins in the pull down and in the input were determined using anti-his antibody. The ponceau staining in the lower panel showed the equal loading of GST tagged proteins. **c**. GST pulldown assay using GST NOD2 LRR with recombinant his-ELMO1 FL or his-ELMO1 CT followed by immunoblotting using anti-His antibody. The ponceau staining in the lower panel showed the equal loading of GST tagged proteins. The densitometry of the pulldowns shown in the graph is from three independent experiments where the mann whitney U test showed the p value of<0.01 represented as **.

Previously, we have demonstrated that ELMO1 is involved in microbial sensing and induction of intestinal inflammation [6]. Both ELMO1 and NOD2 are bacterial sensor proteins that play a role in autophagy and facilitate bacterial degradation [8,22]. Since ELMO1 and NOD2 regulate intestinal immune response against bacterial infections [6–8,16,23–25], we assessed if ELMO1 and NOD2 could interact physiologically. We transiently expressed HA-tagged NOD2, and Flag-tagged ELMO1 in HEK293 cells and performed co-immunoprecipitation. Immunoprecipitation of ELMO1 with FLAG antibody showed that ELMO1 can interact with NOD2 (Figure 1(a)). To determine the interacting domains between ELMO1 and NOD2, GST-ELMO1 full length (FL) and GST-ELMO1 C-terminal domain (CT) were immobilized on glutathione beads and GST pulldown assay was performed with recombinant His-NOD2 CARD or His-NOD2 LRR domains. Our data suggests that only NOD2 LRR region could bind ELMO1 and CT domain of ELMO1 is sufficient for this interaction (Figure 1(b)). GST pulldown assay using GST NOD2 LRR with His-ELMO1 FL or His-ELMO1 CT, showed that the CT domain of ELMO1 had higher binding compared to the FL ELMO1 (Figure 1(c)). Higher binding of His-ELMO1-CT was also observed when varying concentrations of GST-NOD2-LRR regions were used for pull down assay (**Figure S1**).

**Figure 2.**
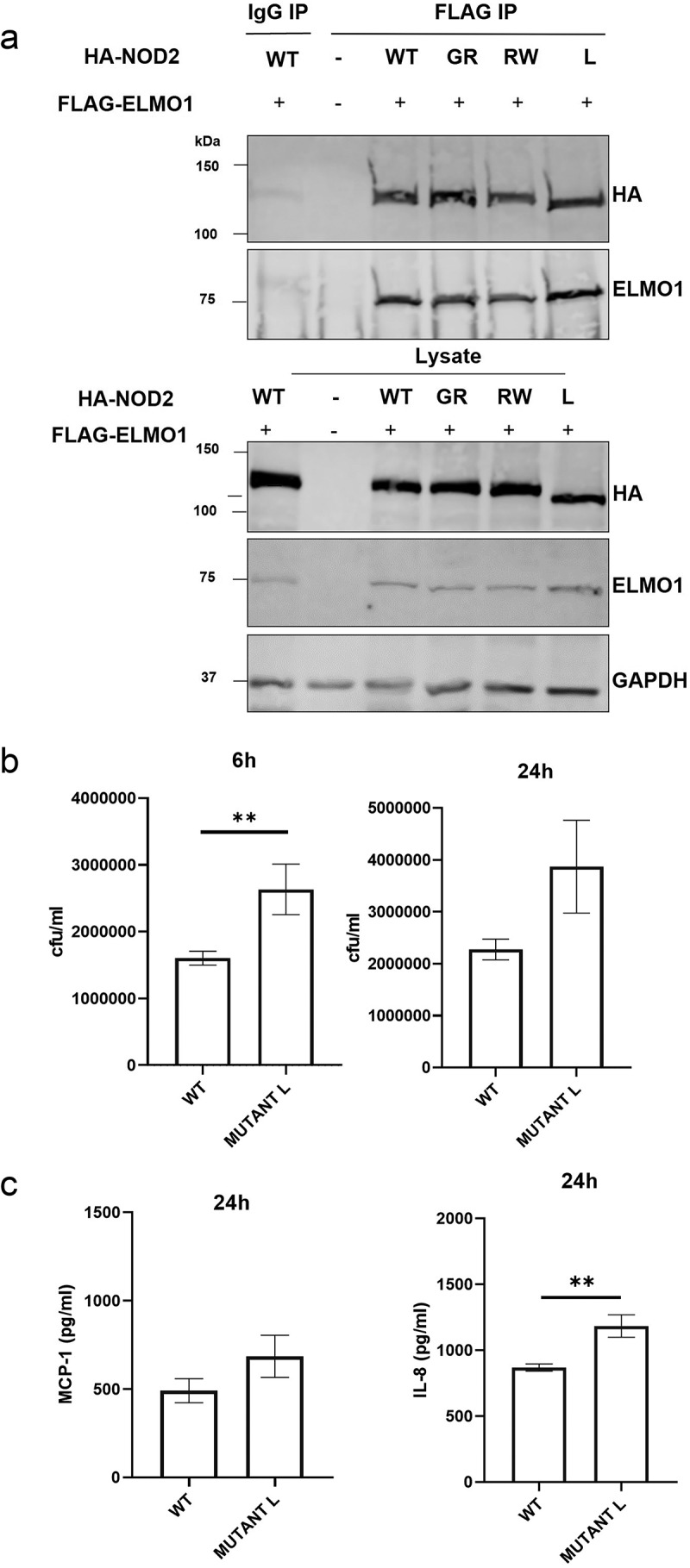
The involvement of NOD2 mutant in bacterial clearance and in inflammation. **a**. HEK293 cells were co-transfected with Flag-ELMO1 and with HA-NOD2 [WT and mutant (GR - G908 R, RW – R702W, L - L1007fs)]. After transfections, cells were lysed, normalized for protein content and precipitated using anti-FLAG antibody. Immunoprecipitants and cell lysates were visualized by immunoblotting with corresponding antibodies. **b**. HEK293 cells were transfected with vectors over-expressing ELMO1 and BAI1, with either NOD2 WT or NOD2L1007fs (Mutant L). Cells were infected with AIEC-LF82 for 6h and gentamicin was used to kill extracellular bacteria. The bacterial count at 6 h and 24 h were plotted from three different experiments. **c**. the supernatant from B at 24 h was used to measure cytokine by ELISA.Results shown are mean ± SEM Mann Whitney test showed p value of < 0.01 represented as**.

The LRR domain is required for binding to MDP, which results in unfolding of NOD2 from auto-inhibitory state to its active state [26,27]. Since most implicated mutations in NOD2 are present within and around the LRR domain, we next evaluated the interaction of ELMO1 with selected NOD2 mutants (GR - G908R, RW – R702W, L – L1007fs), that are associated with susceptibility to CD [19,20]. HEK293 cells were transiently transfected with FLAG-ELMO1 and HA-NOD2 WT (Wild Type) or HA-NOD2 mutants (GR - G908 R, RW – R702W, L – L1007fs) followed by co-immunoprecipitation with FLAG antibody. Our results showed that there were no significant differences in the binding of NOD2 mutants with ELMO1 when compared to NOD2 WT (Figure 2(a)). This data indicated that these CD- associated NOD2 mutations did not alter the interaction of NOD2 with ELMO1.

To further understand the physiological relevance of this interaction, we co-transfected HEK293 cells with wild type NOD2 plasmid or NOD2 L1007fsinsC (L1007fs or mutant L) mutant in the presence of ELMO1 and BAI1. We used the L1007fs mutant as this frameshift mutation is most common in CD patients. We found higher bacterial load of *AIEC*-LF82 infection in mutant L compared to WT at two different time points (6 h and 24 h post infection) (Figure 2(b)). Interestingly, the mutant L was also associated with higher levels of pro-inflammatory cytokines – IL-8 and MCP-1 after 24 h of infection with *AIEC*-LF82 (Figure 2(c)). Pathogens are known to reduce host NF-kB activity in order to enhance their entry by decreasing pro-inflammatory cytokines [28,29]. NFkB activity measured by luciferase reporter assay showed lower NF-kB activity in the mutant L compared to WT (**Figure S2A**). MDP is the ligand for NOD2 activation, which leads to IL-8 production, so when HEK293 cells were treated with MDP for 6 h, reduced levels of IL-8 were observed in the mutant L as compared to WT (**Figure S2B**). Collectively, our data showed that mutation in NOD2 does not affect its interaction with ELMO1 however it was associated with increased bacterial burden and modulated immune response.

### ELMO1-NOD2 interaction fine-tunes paracellular permeability and bacterial load in the epithelium

**Figure 3.**
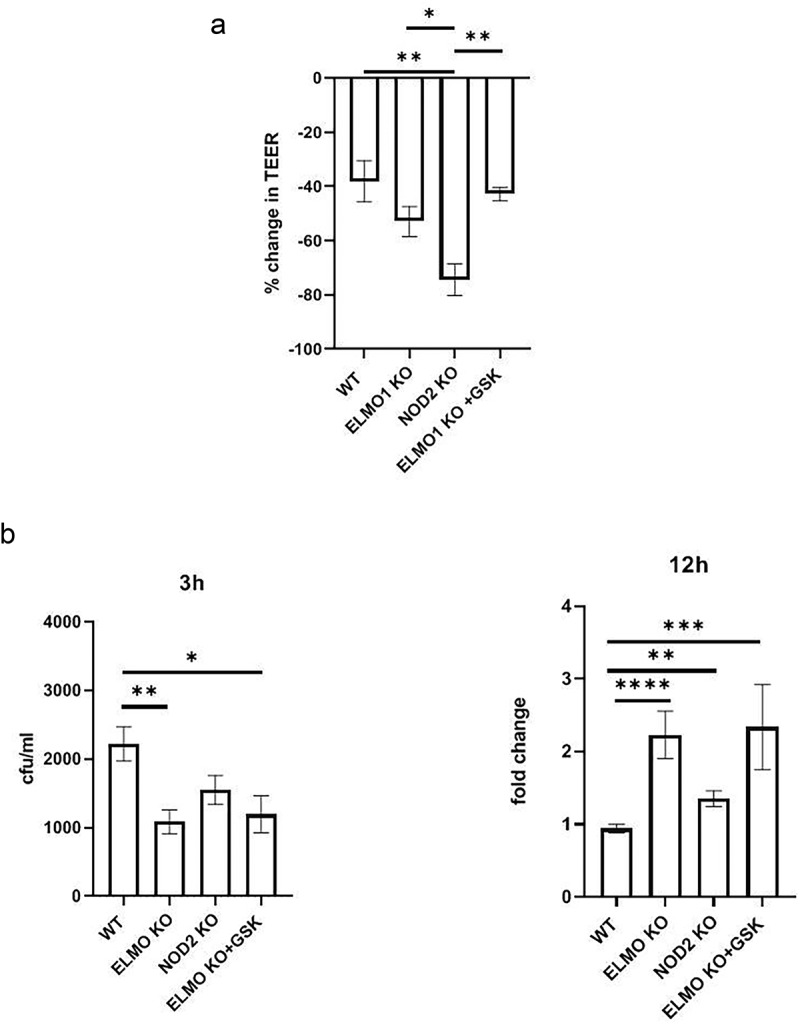
**The impact of ELMO1 and NOD2 in bacterial entry and viability in murine ileal EDMs. a**. Enteroid-derived monolayers (EDMs) from age and gender matched WT, NOD2 KO and ELMO1 KO mouse were infected with *AIEC*-LF82 for 8 h. GSK717, a pharmacologic inhibitor of NOD2 was used in ELMO1 KO EDMs to inhibit NOD2 signalling. Transepithelial electrical resistance (TEER) was measured at time intervals and plotted as percentage change at 8 h compared to 0 h. **b**. the same EDMs from (a) were infected with *AIEC*-LF82; the bacterial internalization determined at 3 h has been plotted as colony-forming units (cfu/mL) and bacterial survival was measured at 12 h where the fold change was calculated considering WT EDMs as 1.Results shown are mean ± SEM as determined by Mann–Whitney U test. *p value is* considered significant if the value < 0.05, < 0.01 < 0.001 and < 0.0001 represented as *, **, *** and **** respectively.

An intact epithelial barrier spatially segregates luminal microbes and protects the host from invasion and dissemination of these microbes. There is substantial evidence that NOD2 regulates intestinal barrier function through myosin light chain kinase (MLCK) activity and mutations in NOD2 causes barrier defects in mice [30]. To assess the impact of NOD2 on the integrity of gut barrier following infection, we challenged EDMs from WT, ELMO1 KO and NOD2 KO mice with *AIEC*-LF82 infection and then assessed the gut barrier integrity by measuring the transepithelial electrical resistance (TEER). We found that NOD2 KO cells had higher paracellular permeability and low resistance to flow-through bacteria on apical side compared to WT cells after 8 h of *AIEC*-LF82 infection (Figure 3(a)). There was no significant difference in TEER values of ELMO1 deficient cells compared to WT cells. Next, to inhibit NOD2 signalling in ELMO1 KO EDMs, we used a potent small molecule NOD2 inhibitor, GSK717. Interestingly, ELMO1 KO EDMs treated with GSK717 showed reduced paracellular permeability with high resistance compared to NOD2 KO EDMs (Figure 3(a)) suggesting that NOD2 is essential for gut barrier integrity.

We previously showed that ELMO1 regulates bacterial entry in intestinal cells, so we further assessed the impact of interaction of ELMO1 and NOD2 in bacterial internalization and survival in intestinal epithelium. We infected murine ileum EDMs with *AIEC-*LF82 and assessed bacterial entry and survival. Since mouse EDMs lack CEACAM receptors needed specifically by *AIEC*-LF82 for invasion into intestinal cells, the number of internalized bacteria could be low (Figure 3(b)). Like our previous study [11] and as shown inFigure 3(b), ELMO1 KO ileum EDMs had lower number of internalized bacteria after 3h of infection, as compared to both WT and NOD2 KO ileum EDMs. Although a smaller number of bacteria entered in ELMO1 KO cells, the bacterial count was higher at 12 h of infection as compared to WT ileum EDMs, depicting prolonged survival and delayed clearance. Similar to ELMO1 deficient EDMs, NOD2 KO EDMs and ELMO1 KO EDMs treated with GSK717 also had a defect in bacterial internalization, and a delayed clearance of bacteria thus leading to higher load after 12 h of infection (Figure 3(b)).

We have previously shown that ELMO1 present in phagosome regulates bacterial clearance in macrophages [8,13]. Further, ELMO1 KO mice showed reduced colonic inflammation and pro-inflammatory cytokines following intestinal pathogen infection [6,9]. Further, to substantiate our findings in murine EDMs we evaluated the immune response in bone marrow-derived macrophages (BMDM) isolated from NOD2 KO mice. We found that the secretion of pro-inflammatory cytokine KC and TNF in BMDMs were low in NOD2 KO mice compared to WT when infected with LF82 or treated with MDP. (Figure S3A-C). Also, the bacterial burden in BMDMs from NOD2 KO mice was significantly higher compared to WT (Figure S3D).

### ELMO1-NOD2 interaction in macrophages regulates bacterial survival, immune responses, and ROS in macrophages

**Figure 4.**
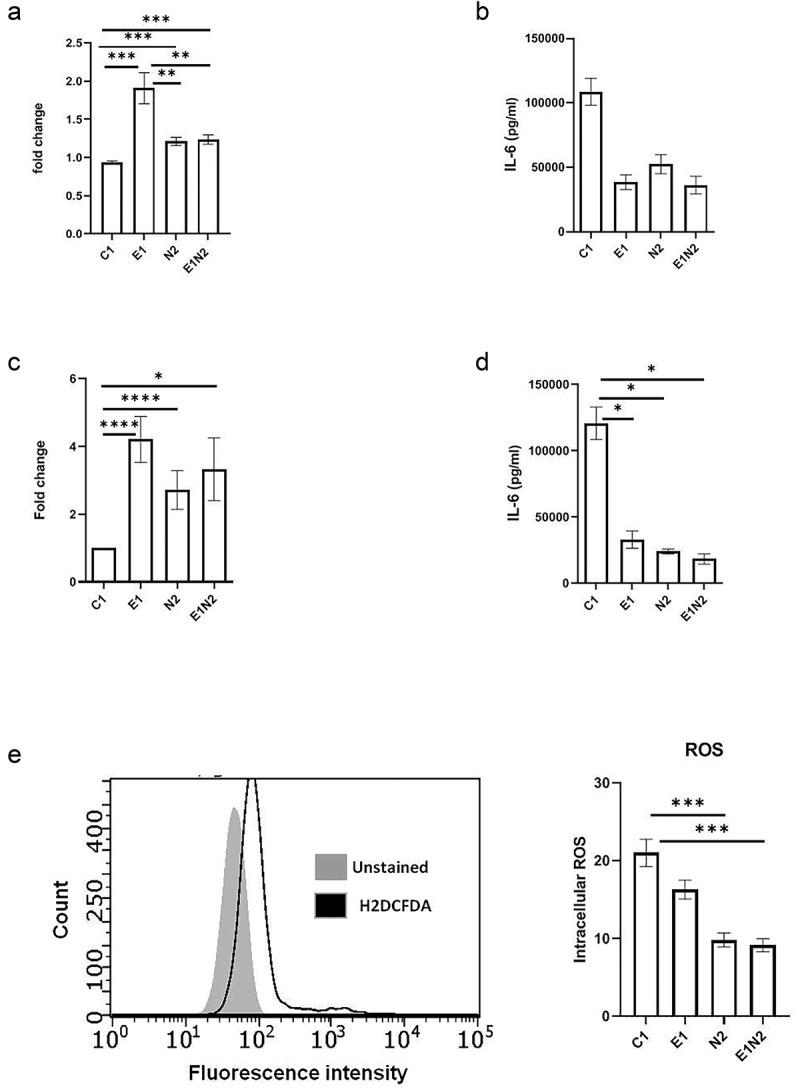
The impact of ELMO1-NOD2 interaction in bacterial clearance and inflammation in macrophages after AIEC-LF82 infection. **a**. J774 cells were infected with *AIEC*-LF82 for 1 h, after which extracellular bacteria were killed by gentamicin treatment. Cells were incubated for 12 h, then lysed, serially diluted, and plated for colony-forming units (cfu). For bacterial survival at 12 h, the cfu at 12 h were normalized to the cfu of bacterial entry at 1 h for each of the respective macrophages. The graph represents the fold change in the bacterial survival, at 12 h calculated by considering bacterial survival of C1 as 1 and the relative bacterial survival in other cells are compared to C1. **b**. The level of pro-inflammatory cytokines (IL-6) secreted in supernatants collected at end of 12 h from “A.” **c**. Murine macrophages were exposed to SL1344 for 1 h, after which extracellular bacteria were killed by gentamicin treatment. Cells were incubated for 12 h, then lysed, serially diluted, and plated for cfu. For bacterial survival at 12 h, the cfu at 12 h were normalized to the cfu of bacterial entry at 0 min for each of the respective macrophages. The graph represents the fold change in the bacterial survival, at 12 h calculated by considering bacterial survival of C1 as 1 and the relative bacterial survival in other cells are compared to C1. **d**. the level of pro-inflammatory cytokines (IL-6) secreted in supernatants collected at end of 12 h from “C.” **e**. J774 macrophages were infected with *AIEC*-LF82 infection for 30 min, followed by treatment with high gentamicin and 1 µM H2DCFDA for 60 min. Cells were then washed and analysed on a flow cytometer for detection of total cellular ROS. Bar graph on right show percent of cells expressing ROS, data is displayed as mean ± SEM. Graph shows transcript levels after infection.Results shown are mean ± SEM as determined by Mann–Whitney U test. *p* value is considered significant if the value < 0.05, < 0.01 < 0.001 and < 0.0001 represented as *, **, *** and **** respectively.

To investigate the crosstalk between ELMO1 and NOD2 in macrophages, we used lentiviral vectors expressing shRNA to knockdown either ELMO1(E1), NOD2(N2), or both (E1N2) in murine macrophages and compared them to macrophages with control shRNA (C1). As depicted in Figure S4A, NOD2 shRNA resulted in knockdown of *NOD2* transcript in both N2 and E1N2 cells. The downregulation of ELMO1 in E1 and E1N2 cells were confirmed by western blot (Figure S4B). Bacterial sensing is critical in the generation of immune response. Since both NOD2 and ELMO1 are bacterial sensors, we further investigated the effect of their knockdown on bacterial, survival, and induction of innate immune response in macrophages. We infected C1, E1, N2 and E1N2 macrophages with *AIEC-*LF82 and evaluated bacterial burden. We observed that the absence of either or both of the proteins resulted in delayed bacterial clearance which led to higher bacterial load compared to control cells (Figure 4(a)). Previously, we had reported that ELMO1 regulated the immune response against infection in macrophages by producing pro-inflammatory cytokines [6,9]. Similarly, to evaluate the influence of ELMO1 and NOD2 interaction, we quantified the levels of pro-inflammatory cytokines in macrophages upon infection with CD-associated *AIEC*-LF82. In *AIEC*-LF82 infection, the absence of either ELMO1 or NOD2 or both resulted in a significant decline in IL-6 levels compared to C1 cells (Figure 4(b)).

We also validated the above results using *Salmonella enterica* serovar Typhimurium strain SL1344 as a model for enteric infection. As observed for *AIEC*-LF82, in case of *Salmonella* infection the absence of either ELMO1 or NOD2 or both resulted in a delayed bacterial clearance (Figure 4(c)) and significant decline in levels of IL-6 (Figure 4(d)) and IL-1β (Figure S5A) compared to C1 cells. Taken together, these results showed that ELMO1 and NOD2 are required for the clearance of *AIEC*-LF82 and *Salmonella* and induction of innate immune response in the immune cells.

ROS production is a natural anti-bacterial response in macrophages against almost all microbes, which reduces the bacterial survival [31]. But some microbes modulate this effect in order to survive inside the host cells. Since we observed higher bacterial survival in absence of ELMO1 or NOD2 or both, we assessed the ROS levels in these cells. We found lower ROS production in E1, N2 and E1N2 cells compared to C1 cells, which shows that the anti-immune response in macrophages was hijacked by microbes in absence of bacterial sensors (Figure 4(e)). To further confirm this, we isolated peritoneal macrophages from WT, Heterozygous ELMO1(Het) and ELMO1 KO mice and infected with LF82 followed by measurement of ROS. Again, we found lower ROS levels in ELMO1 KO mouse as compared to WT or Het mouse (Figure S5B). To confirm if high bacterial load is a consequence of low ROS release in macrophages (C1, E1, N2, E1N2), we used ROS scavenger; N-acetyl cysteine (NAC) which reduces ROS levels. We found higher bacterial survival in *AIEC*-LF82 infected cells treated with NAC compared to untreated cells (Figure S5C). However, there was no significant difference in bacterial levels among ELMO1 KO and NOD2 KO cells upon addition of NAC as the ROS level in these cells were already very low. This result indicated that lower ROS levels in ELMO1 and NOD2 deficient cells are responsible for delayed bacterial clearance.

## Discussion

Bacterial sensing is considered to be the rate-limiting step in combating any infection. Impaired bacterial sensing has been implicated as the cause of several auto-immune and inflammatory diseases, including CD [32,33]. In the present study, we have reported for the first time that there is direct interaction of two important microbial sensing proteins, NOD2 and ELMO1, which plays a significant role in determining host response to pathogens. The salient features of the study are: 1. C terminal region of ELMO1 is sufficient for interaction with NOD2; 2. LRR region of NOD2 is involved in binding to ELMO1; 3. The absence of either or both the proteins results in dysregulated antibacterial response in case of *AIEC*-LF82 and *Salmonella* infection.

The C terminal region of ELMO1 is involved in the interaction of DOCK 180 through its PH domain, resulting in the regulation of Rac, ensuing bacterial engulfment and immune response [34]. In addition, the C-terminal of ELMO1 is involved in binding with bacterial effectors with a signature WxxxE motif resulting in differential immune response between pathogens and commensals [9]. Previously we had shown that BAI1 acts as PRR and identifies the core component of LPS [10]. The LRR region of NOD2 is similarly involved in the recognition of bacterial components and has been identified to bind to bacterial cell wall component MDP [12]. In addition, mutations in NOD2 are associated with delayed bacterial clearance in CD patients [32]. Therefore, the interaction between ELMO1 and LRR region of NOD2 implies coordination between two different bacterial sensing systems, which could affect bacterial recognition, bacterial engulfment and clearance, and regulation of the immune response. In our previous study we have identified ELMO1/MCP1 axis in epithelial cell and immune cells for triggering inflammatory response in IBD [11]. Herein, we studied the effect of ELMO1-NOD2 interaction on bacterial pathogenesis in both epithelial and immune cells.

Mutations in the LRR region of NOD2 have been associated with CD [19,20]. In this study, we found that although the mutations do not affect the interaction between ELMO1 and NOD2, they affect the clearance of bacteria. Our studies using HEK293 cell lines and mutant NOD2 have substantiated that the interaction of ELMO1-NOD2 does not affect the internalization of bacteria; however, the subsequent immune signalling is dysregulated. L1007fsinsC mutation has been associated with CD and results in the production of truncated NOD2 protein. L1007fsinsC NOD2 mutant has been previously reported to decrease the NFkB activity compared to wild-type NOD2 [19]. As shown in the previous study [19], our study also confirmed the lower NFkB activity in L1007fsinsC mutant, which probably resulted in impaired clearance of bacteria.

*In vivo* and *in vitro* studies have demonstrated the role of NOD2 in maintaining permeability in epithelial cells [35,36]. In a parallel line, our study using EDM models has shown that the absence of NOD2 decreases the barrier integrity resulting in a higher drop in transepithelial electrical resistance. However, a similar effect was not observed in case of ELMO KO and GSK treated cells suggesting that only NOD2 is vital for gut barrier integrity.

Both ELMO1 and NOD2 are involved in bacterial clearance. ELMO1 plays a role in the engulfment, pathogenesis, and immune responses against enteric bacteria [6,9]. ELMO1 has also been associated with LC3 associated phagocytosis, induction of inflammatory cytokines and clearance of bacteria and defects in ELMO1 expression results in reduced clearance of bacteria [8]. NOD2 has been reported to play a role in autophagy, ROS generation, regulation of cytokines and itself can act as antibacterial agent [12–15]. Defects in NOD2 expression or absence of NOD2 can hence result in delayed clearance of microbes [32]. Herein, we assessed if the interaction between ELMO1 and NOD2 could affect the clearance of bacteria in both epithelium and macrophage levels. Epithelial monolayers lacking either or both proteins resulted in higher bacterial load (i.e. delayed clearance). Similarly, higher bacterial loads were recorded in macrophages depleted with either ELMO1 or NOD2 or both compared to control macrophages. Collectively, these findings showed that ELMO1, NOD2, and their interaction are important in bacterial clearance and pathogenesis during *AIEC*-LF82 and *Salmonella* infection. Future studies are needed to assess if the interaction between ELMO1 and NOD2 is crucial in the pathogenesis of other enteric pathogens such as *Shigella, Yersinia*, other strains of *E. coli*.

To investigate the mechanisms of bacterial survival in these cells, we assessed inflammatory cytokines and ROS levels in these cells. As expected, depletion of ELMO1 and/or NOD2 resulted in diminished inflammatory immune responses as shown by reduced level of inflammatory cytokines following the challenge with enteric pathogens. In a parallel line, the level of ROS was reduced in absence of either or both the protein compared to C1 cells. (Figure 4(e)).

Although this study is the first report that shows the interaction between two cytosolic microbial sensors and the relevance of this interaction on enteric infections; our study has some limitations. First, the mechanistic details of ELMO1 and NOD2 interactions are unknown. Therefore, it is difficult to predict the outcomes of this interaction in another bacterial pathogenesis. The impact of this interaction could be additive, synergistic, antagonistic, and/or no effect depending on the cell type, bacteria used and the activated signalling pathway. Second, this study lacks the *in vivo* animal work that could validate the findings further. Finally, the colocalization of ELMO1 and NOD2 using other techniques such as IF and IHC should be considered for future studies.

The result of the present study reveals that NOD2 and ELMO1 can interact with each other directly and can influence the course of bacterial infection by regulating bacterial survival/clearance, ROS generation and immune response during *AIEC*-LF82 and *Salmonella* infection. Further studies are required to explore the structural and molecular details of this interaction and the subsequent pathways involved. Such studies will provide alternative target for therapeutics in case of chronic inflammatory diseases such as Crohn’s disease where defective sensing of luminal bacteria in predisposed genetic background contribute to the disease pathogenesis.

## Materials and methods

### Animals

WT, ELMO1^−/−^, and NOD2^−/−^ C57BL/6 mice were purchased from Jackson Laboratories. Our animal protocol (ID S18086) used for mice experiments was approved by the UCSD Institutional Animal Care and Use Committee (IACUC) policies

### Bacterial strains

*Escherichia coli* strain LF82 and *Salmonella enterica* Typhimurium SL1344 were either isolated or purchased and maintained as mentioned before [9,11,37]. Bacterial load used in the infection experiments was determined as a multiplicity of infection (MOI) which is 1:10 for macrophages, 1:30 for EDMs, and 1:100 for HEKs.

### Cell lines

HEK293 cells and J774 macrophage cells (J774) cells were maintained in high glucose DMEM as described before [6]. Lipofectamine 2000 (Invitrogen) was used to transfect plasmids inside the cells as mentioned in manufacturer’s protocol.

### shRNA lentiviral transduction

NOD2 MISSION shRNA Lentiviral Transduction Particles from Sigma Aldrich (TRCN0000066813) were used to stably down-regulate NOD2 in J774 macrophage. J774 cells were seeded in 96-well plates at 1.6 × 10^4 cells per well for 24 hrs. Cell media supplemented with 8 ug/ml Polybrene (Sigma Aldrich, St Louis MO) was added to cells followed by 10ul of lentivirus particles (4.7 × 10^7 VP/mL titre value). Next day, the media containing lentiviral particles was removed from wells and fresh media was added. For selection, 1 mg/ml G418 (Cat# G8168, Sigma Aldrich) was added to cells 48 h after lentivirus transduction. Cell media was changed every 2–3 days with fresh G418- containing media until resistant colonies were identified. RNA was extracted to determine knockdown efficacy and cell lines were kept under selective pressure using G418 containing media.

### Development of 3D enteroids and enteroid-derived monolayers (EDMs) from the mouse gut

Stem cells were isolated from the mouse gut as described before [11,38–42]. These cells were organized and expanded as 3D organoids in the presence of basement matrix and WNT containing media [42]. 3D enteriods were digested using tryspin and single cells were plated into a transwell in the presence of 5% conditioned media to differentiate into intestine specific epithelial cells. For all functional assays, experiments were performed multiple times with EDMs-derived from enteroids collected from at least three different mice, including both the genders. The transepithelial electrical resistance (TEER) was measured in the EDMs using Epithelial Volt/Ohm (TEER) Meter [38].

### Isolation of bone marrow – derived macrophages

BMDMs were isolated following the protocol described elsewhere [6,9,10,43,44]. Briefly, femur bones were collected from C57B6 mice after euthanization. The bone marrow cells were then flushed from the femur bones using 5 G needle and RPMI medium. The cells were then centrifuged, and the RBCs were lysed by incubating with 1× RBCs lysis buffer (Thermo Fisher Scientific) for 3 minutes. The remaining bone marrow cells were precipitated and resuspended in DMEM media containing 10% FBS, 20% LCCM (L929 cells conditioned media), and ciprofloxacin (0 µg/ml) and incubated at 37°C. After 3 days, the media was replaced with new media devoid of antibiotics.

### Assessment of bacterial burden using gentamicin protection assay

WT, ELMO1^−/−^ and NOD2^−/−^ 3D organoids were trypsinized and about 3 × 10^5 cells were used to prepare enteroid derived monolayer (EDMs). EDMs were infected with *AIEC-*LF82 (MOI 10) and then the bacterial load was determined after 2 h of infection of as described before [6,9,10]. For transfected HEKs, approximately 4 × 10^5 cells were plated 6 h prior before infection and bacterial count was determined after 6h following infection. After infection cells were lysed with 1% TritonX-100, followed by serial dilution and plating on LB agar as done previously.

### Measurement the level of inflammatory cytokines by ELISA

Control (C1), ELMO1 (E1)/NOD2 (N2), or (E1N2)- knock down J774 cells were infected or not with *AIEC*-LF82/*Salmonella enterica* Typhimurium SL1344. Supernatants were collected and assessed for different cytokines such as IL-6, IL-1β, IL8, and MCP1 using the ELISA kit (R&D systems).

### Gene expression by qRT-PCR

To assess 5”UTRNOD2 transcript expression, we used Quick-RNA Miniprep Kit (ZymoResearch) to extract RNA, followed by cDNA preparation (Quantabio), and running qRT-PCR (SYBR green, Bimake). The relative expression of 5”UTRNOD2 gene was determined by calculating ∆∆Ct (Ct of 5”UTRNOD2 -Ct of housekeeping gene). The primers used were as follows: 5”UTRNOD2 Forward 5”GGACCTGGACTCCTCCAAA3” and Reverse 5”GCTGGGCTGAGAACACATAG3.”

### Expression constructs

NOD2 mutant plasmids (HA-NOD2 R702W, HA-NOD2 G908 R and HA-NOD2 L1007fs) were generated by site directed mutagenesis on HA-NOD2 plasmid (a gift from Dana Philpott) using the QuickChange II Site-Directed Mutagenesis kit (Agilent Technologies). The CARD domain (amino acids 28–265) and LRR domain (amino acids 744–1040) of NOD2 were generated by PCR and cloned into pET-28a (+) plasmid vector (Novagene). All plasmid constructs and mutagenesis were verified by sequencing and protein expression was verified by western blot analysis.

### Immunoprecipitation and western blotting

Transfected HEK293 cells were lysed in NP buffer and the protein content was measured by the lowry assay. Then the protein lysate (about 1 mg) was incubated with 40 µl of Ezview Red ANTI-FLAG M2 Affinity Gel (Cat # F2426-1ML, Sigma-Aldrich) overnight at 4°C. Washing of beads was repeated 4 times using NP lysis buffer at 1500 rpm for 2mins. Immunoprecipitates were eluted from the beads by resuspending beads in 0 μl of 2× SDS-PAGE sample buffer and boiled for 0 mins at 100°C. Proteins were separated by running on a SDS-PAGE protein gel, followed by transfer onto Immobilon-P PVDF membrane. The membrane was immunoblotted with primary antibodies followed by secondary antibody incubation either anti-mouse IgG HRP-linked Antibody (Cat # 7076S, Cell Signaling Tech) or anti-rabbit IgG HRP-linked antibody (Cat #7074S, Cell Signalling Tech). Protein bands were detected using ECL (Amersham Biosciences). Western blot was analysed using the following primary antibodies: Mouse monoclonal anti-poly-HIS (1:500) (H1029, Sigma-Aldrich), Rabbit monoclonal GST (91G1) (1:1000) (2625S, Cell Signalling), Mouse monoclonal Flag (9A3) (1:1000) (8146S, Cell Signalling), Mouse monoclonal Elmo1 (B-7) (1:500) (Cat sc -271,519, Santa Cruz Biotechnology), IRDye 800CW Goat anti-Mouse IgG Secondary (1:10,000) (926–32210, LI-COR Biosciences), IRDye 680RD Goat anti-Rabbit IgG Secondary (1:10,000) (926–68071, LI-COR Biosciences), HA -Tag (C29F4) Rabbit mAb (3724S, Cell Signalling), Tubulin Antibody (2144S, Cell Signalling), GAPDH (14C10) Rabbit mAb (2118S, Cell Signalling).

### Purification of GST-NOD2-LRR

The bacterial colony (*Escherichia coli* BL21 expressing GST-NOD2-LRR) was picked from a freshly streaked plate, inoculated in 0 ml LB supplemented with 0 µg/ml ampicillin and incubated overnight at 37°C. The next day this 0 ml pre-culture was transferred to 1 L of LB supplemented with 0 µg/ml ampicillin and further incubated at 37°C (shaking at 220 rpm). After the OD of LB at A600 reaches to 0.6, 1 mM isopropyl-β-D-thiogalactoside (IPTG) is added and kept for overnight at 25°C. The cells were harvested by centrifugation for 0 min at 3750 rpm at 4°C. Cell pellet from 1L culture was resuspended in 0 ml of lysis buffer and sonicated in 4°C cold room for 20 s four times, at 2 min intervals. The sonicated cell lysates were centrifuged for 0 min at 12,000 rpm at 4°C. The supernatant was collected and incubated with GST beads for 1h at 4°C on rotator (GE Healthcare). GST beads were washed three times with phosphate buffered saline (PBS), and the attached proteins were eluted by adding reducing sample buffer. The purity of proteins was analysed on 10% SDS-PAGE stained with Coomassie blue, quantified using BSA as standard, aliquoted and stored at −80°C.

### Pulldown assay

Purified GST-NOD2-LRR was immobilized on GST beads for overnight at 4°C. The immobilized GST-NOD2-LRR was incubated with His-ELMO-CT and His-ELMO-FL proteins for binding for 4h at 4°C using the protocol as described before [9]. GST protein was used to detect non-specific binding.

### Determination of intracellular ROS

Approximately 1 million cells were loaded with 5 μM dichlorofluorescin diacetate (H2DCFDA) according to standard procedures [45,46]. The cells were then washed and resuspended before being examined by flow cytometry (Guava® easyCyte Benchtop Flow Cytometer, Millipore). N-Acetyl l-cysteine (NAC) was used as scavenger of ROS at an optimized concentration of 10 µM as we have done before [47].

### Statistics

Results were mainly presented as the mean ± SEM otherwise, they were specifically described. *P values* were determined using non-parametric Mann–Whitney U test, and they considered significant if the values were<0.05.

## Supplementary Material

Supplemental Material

## Data Availability

Data of this study is present in the main text and supplementary materials.
