# Supplementary figures and images for: The crosstalk between microbial sensors ELMO1 and NOD2 shape intestinal immune responses
Source: Virulence. 2023 Feb 19;14(1):2171690. doi: 10.1080/21505594.2023.2171690 (PMC9980453; doi:10.1080/21505594.2023.2171690)

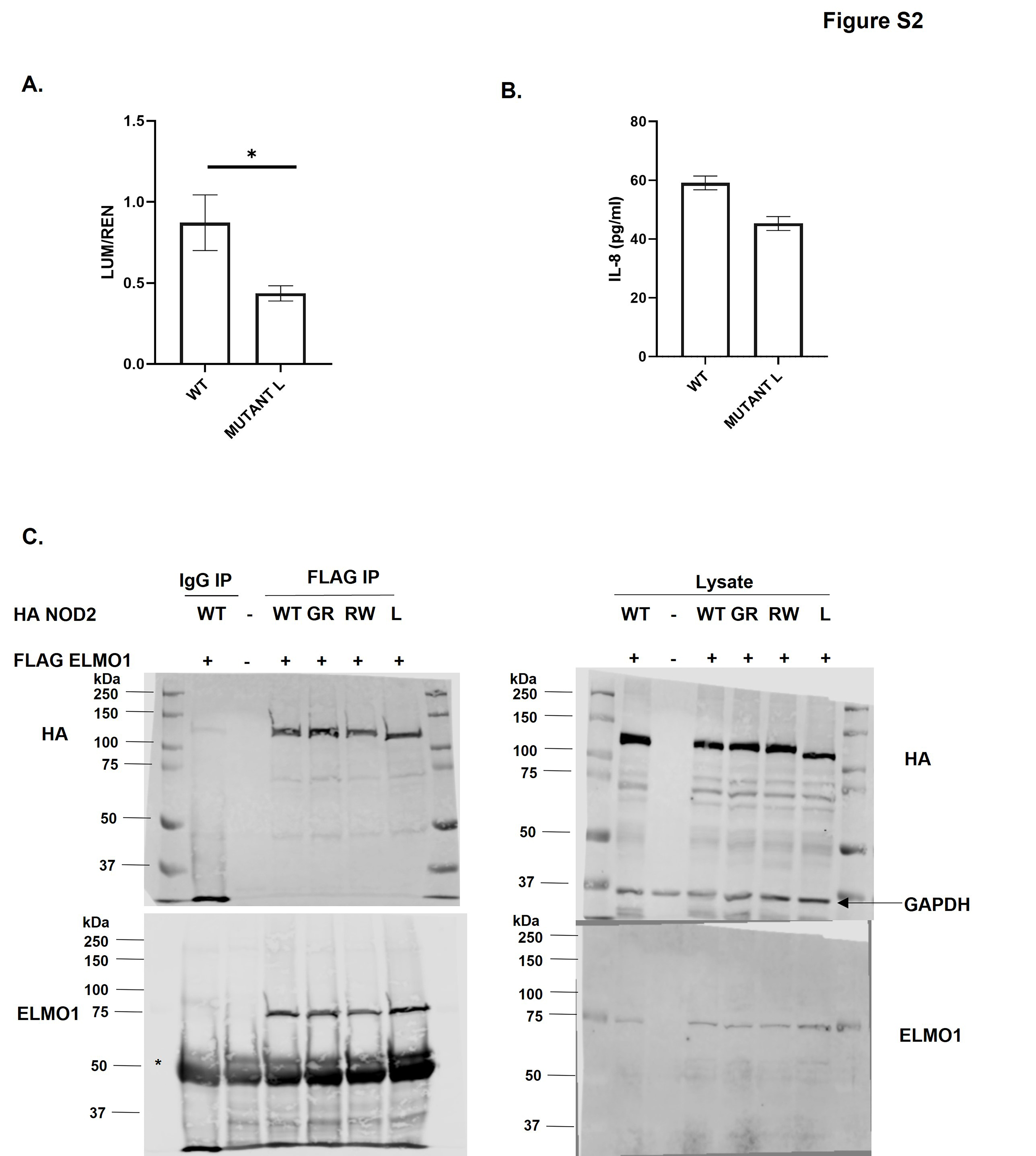

Supplement: Supplemental Material [file KVIR_A_2171690_SM7060.zip › supplementary/FigS2C new.jpg]

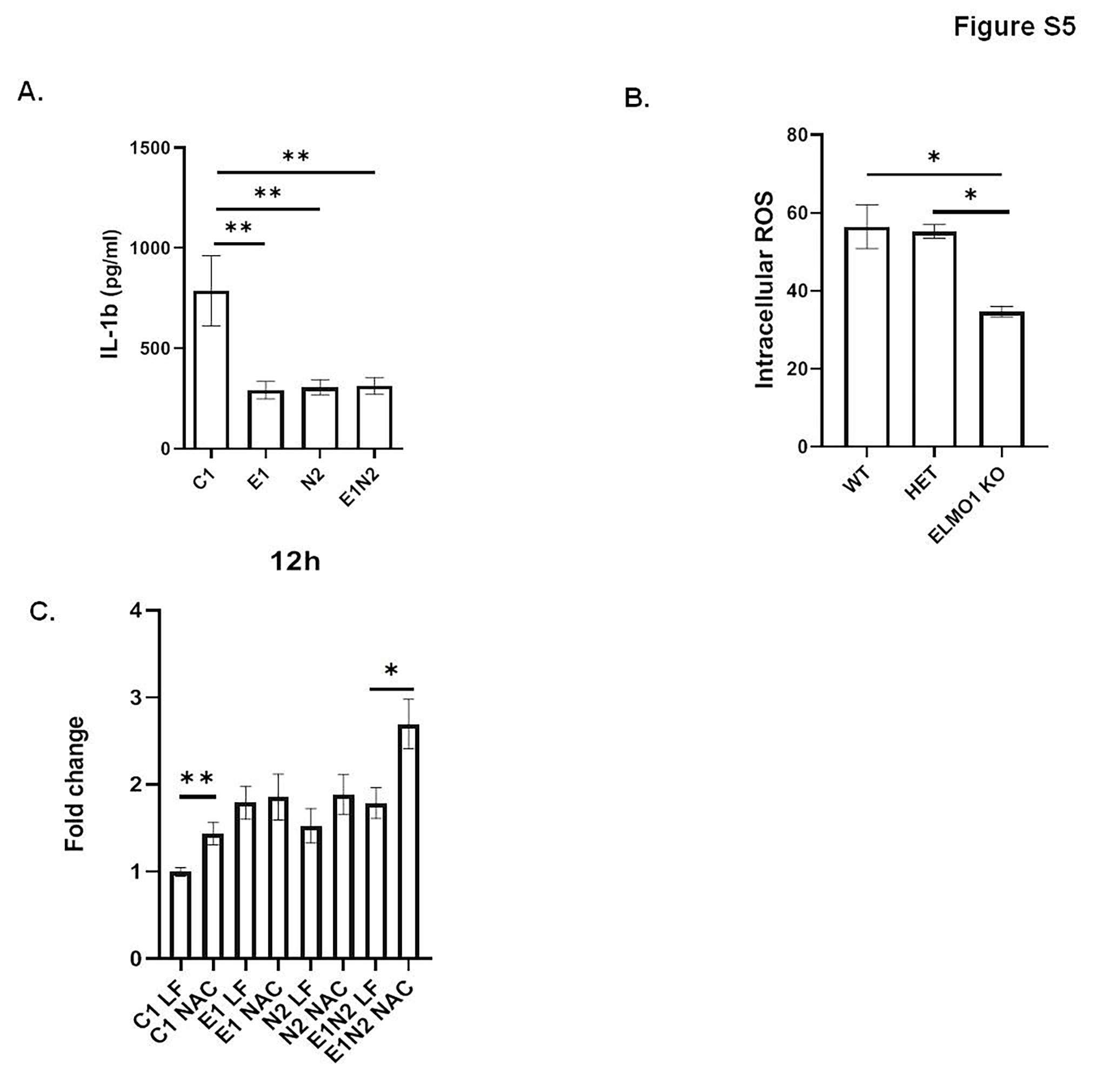

Supplement: Supplemental Material [file KVIR_A_2171690_SM7060.zip › supplementary/Picture S5.jpg]

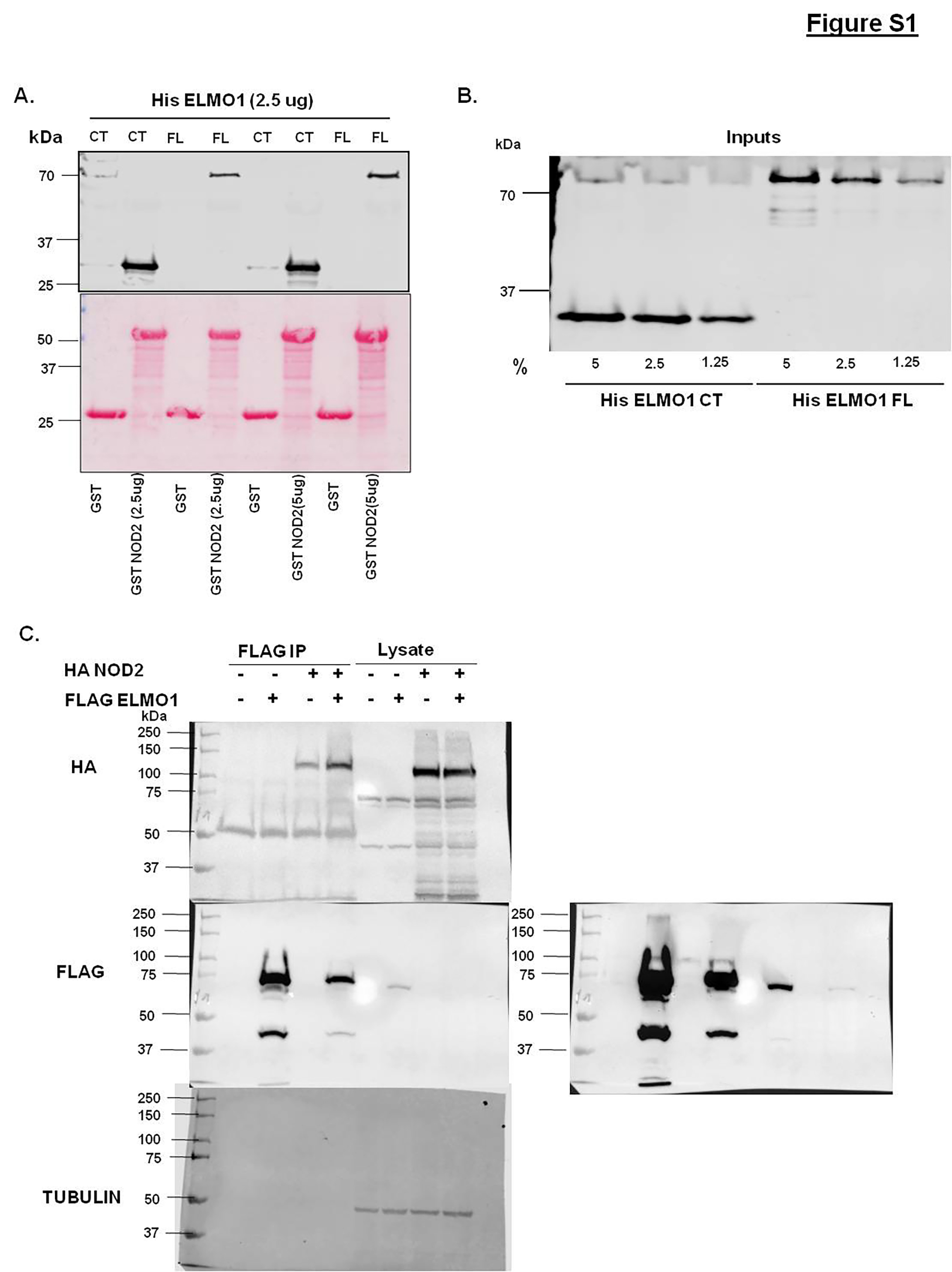

Supplement: Supplemental Material [file KVIR_A_2171690_SM7060.zip › supplementary/PictureS1.jpg]

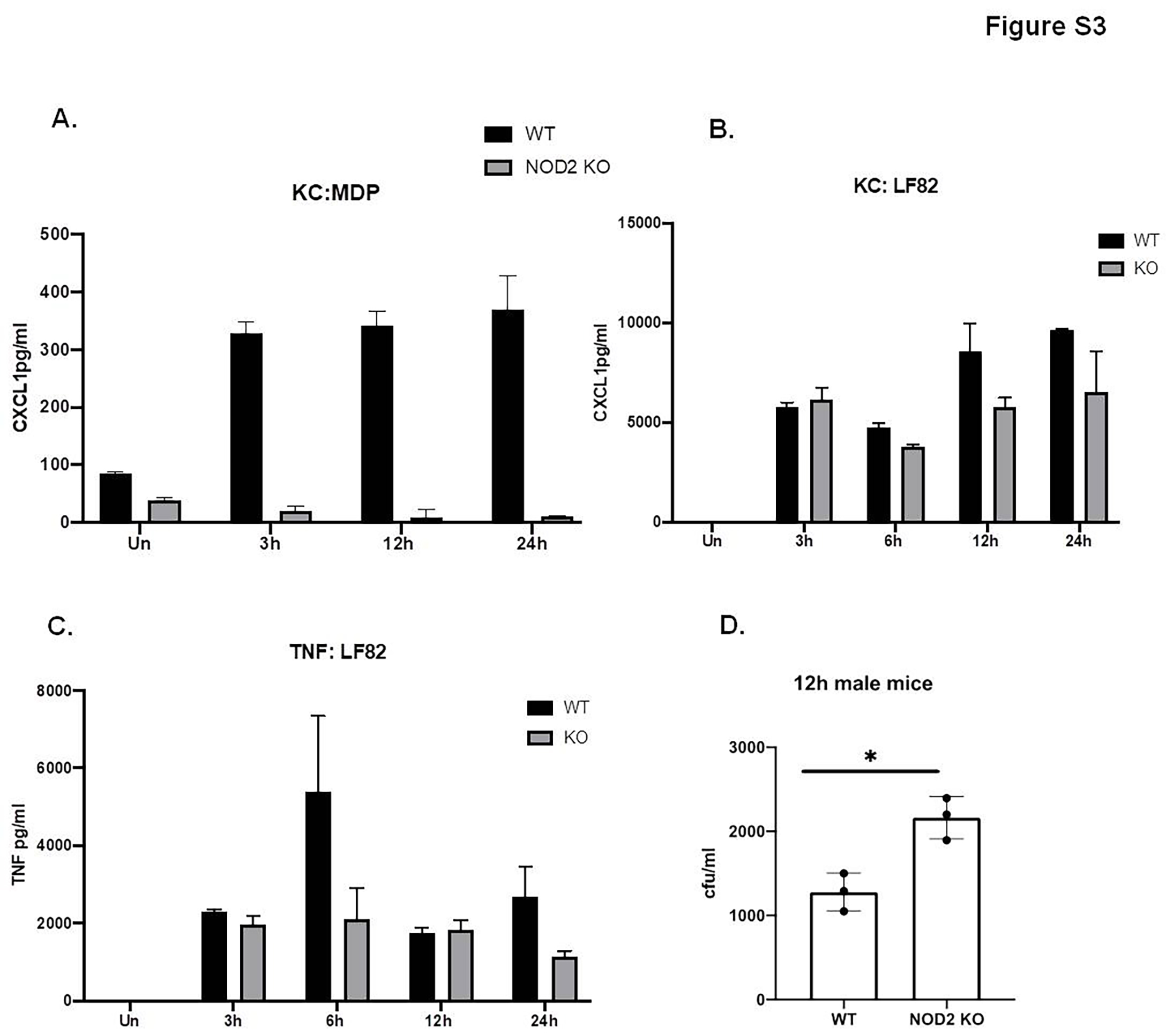

Supplement: Supplemental Material [file KVIR_A_2171690_SM7060.zip › supplementary/Pictures3.jpg]

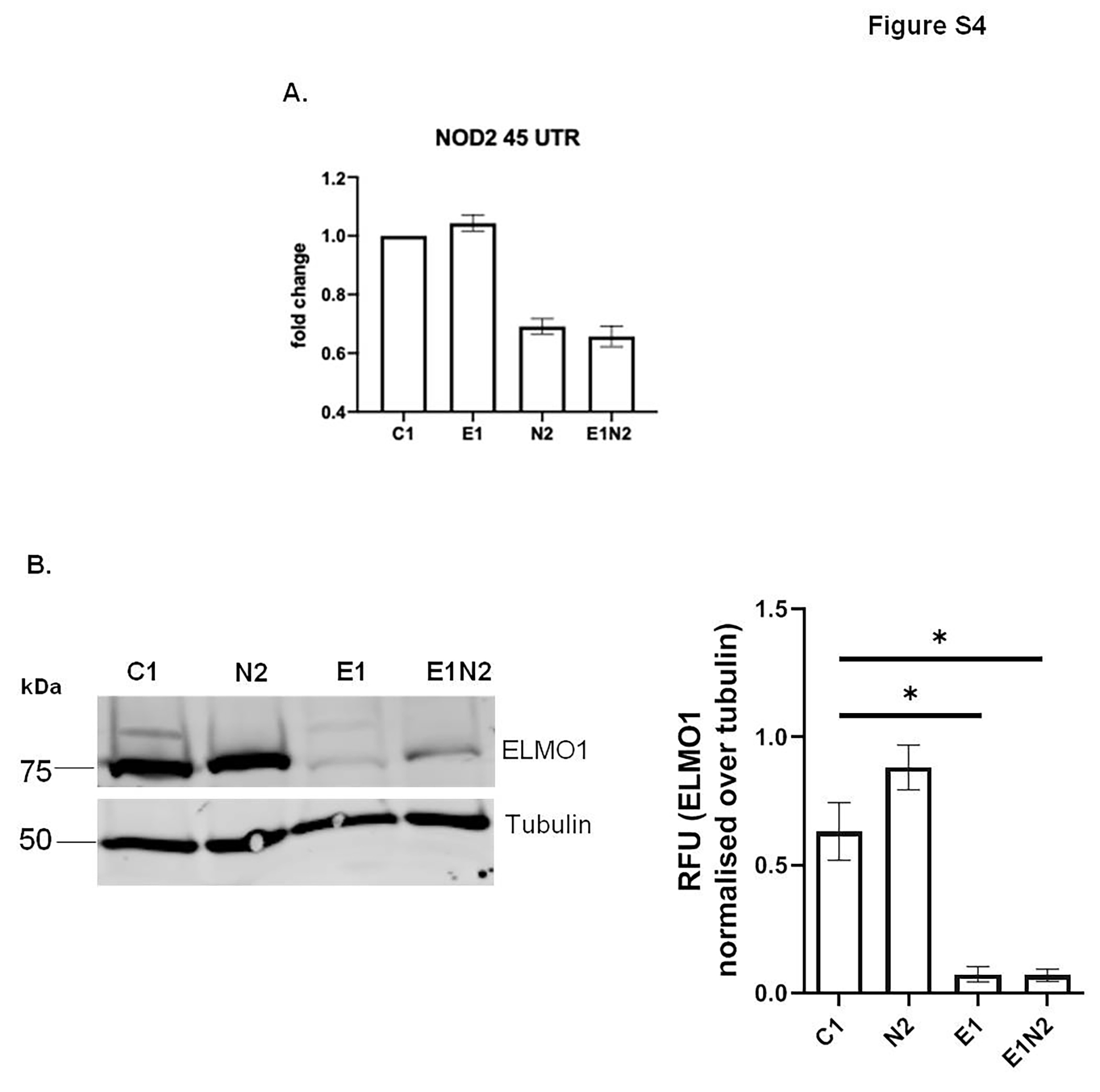

Supplement: Supplemental Material [file KVIR_A_2171690_SM7060.zip › supplementary/PictureS4.jpg]
